# The Real-World Data in Japanese Patients with Unresectable Hepatocellular Carcinoma Treated with Lenvatinib from a Nationwide Multicenter Study

**DOI:** 10.3390/cancers13112608

**Published:** 2021-05-26

**Authors:** Kaoru Tsuchiya, Masayuki Kurosaki, Azusa Sakamoto, Hiroyuki Marusawa, Yuji Kojima, Chitomi Hasebe, Hirotaka Arai, Kouji Joko, Masahiko Kondo, Keiji Tsuji, Tetsuro Sohda, Hiroyuki Kimura, Chikara Ogawa, Yasushi Uchida, Shuichi Wada, Haruhiko Kobashi, Koichiro Furuta, Masaya Shigeno, Atsunori Kusakabe, Takehiro Akahane, Ryoichi Narita, Hideo Yoshida, Akeri Mitsuda, Yasushi Ide, Tomomichi Matsushita, Namiki Izumi, on behalf of Japanese Red Cross Liver Study Group

**Affiliations:** 1 Department of Gastroenterology and Hepatology, Musashino Red Cross Hospital, Tokyo 180-8610, Japan; tsuchiya@musashino.jrc.or.jp (K.T.); kurosaki@musashino.jrc.or.jp (M.K.); 2 Department of Gastroenterology and Hepatology, Japanese Red Cross Osaka Hospital, Osaka 543-855, Japan; azusa-s@osaka-med.jrc.or.jp (A.S.); h.marusawa@osaka-med.jrc.or.jp (H.M.); 3 Department of Gastroenterology and Hepatology, Japanese Red Cross Ise Hospital, Ise 516-8512, Japan; tdim_yrch@ise.jrc.or.jp; 4 Department of Gastroenterology and Hepatology, Japanese Red Cross Asahikawa Hospital, Asahikawa 070-8530, Japan; hasebe@asahikawa-rch.gr.jp; 5 Department of Gastroenterology and Hepatology, Japanese Red Cross Maebashi Hospital, Maebashi 371-0811, Japan; h-arai@maebashi.jrc.or.jp; 6 Department of Gastroenterology and Hepatology, Matsuyama Red Cross Hospital, Matsuyama 790-8524, Japan; koujijoko@matsuyama.jrc.or.jp; 7 Department of Gastroenterology and Hepatology, Japanese Red Cross Otsu Hospital, Otsu 520-0046, Japan; masachan44@gmail.com; 8 Department of Gastroenterology and Hepatology Hiroshima Red Cross Hospital & Atomic-bomb Survivors Hospital, Hiroshima 730-0052, Japan; k-tsuji@hiroshima-med.jrc.or.jp; 9 Department of Hepatology, Japanese Red Cross Fukuoka Hospital, Fukuoka 815-8555, Japan; t-sohda@fukuoka-med.jrc.or.jp; 10 Department of Gastroenterology and Hepatology, Japanese Red Cross Kyoto Daiichi Hospital, Kyoto 605-0981, Japan; hiroyuki-kimura@kyoto1-jrc.org; 11 Department of Gastroenterology and Hepatology, Takamatsu Red Cross Hospital, Takamatsu 760-0017, Japan; chikara.ogawa.19721202@gmail.com; 12 Department of Gastroenterology and Hepatology, Matsue Red Cross Hospital, Matsue 690-0886, Japan; laboratory@matsue.jrc.or.jp; 13 Department of Gastroenterology and Hepatology, Japanese Red Cross Nagano Hospital, Nagano 380-0928, Japan; s.wada@nagano-med.jrc.or.jp; 14 Department of Hepatology, Japanese Red Cross Okayama Hospital, Okayama 770-8607, Japan; kobashi0584@gmail.com; 15 Department of Gastroenterology and Hepatology, Japanese Red Cross Masuda Hospital, Masuda 698-8501, Japan; furuta-k@masuda.jrc.or.jp; 16 Department of Gastroenterology and Hepatology, Japanese Red Cross Nagasaki Genbaku Hospital, Nagasaki 852-8511, Japan; mshi1010@nagasaki-med.jrc.or.jp; 17 Department of Gastroenterology and Hepatology, Japanese Red Cross Nagoya Daini Hospital, Nagoya 466-8650, Japan; akusakabe@nagoya2.jrc.or.jp; 18 Department of Gastroenterology and Hepatology, Japanese Red Cross Ishinomaki Hospital, Ishinomaki 986-8522, Japan; akahane-ttyh@mva.biglobe.ne.jp; 19 Department of Hepatology, Japanese Red Cross Oita Hospital, Oita 870-0033, Japan; narita@oita-rc-hp.jp; 20 Department of Gastroenterology and Hepatology, Japanese Red Cross Medical Center, Tokyo 150-8935, Japan; ch2h-ysd@asahi-net.or.jp; 21 Department of Gastroenterology and Hepatology, Japanese Red Cross Tottori Hospital, Tottori 680-8517, Japan; akeri@tottori-med.jrc.or.jp; 22 Department of Gastroenterology and Hepatology, Japanese Red Cross Karatsu Hospital, Karatsu 847-8588, Japan; ideyasushi@gmail.com; 23 Department of Gastroenterology and Hepatology, Japanese Red Cross Gifu Hospital, Gifu 502-8511, Japan; tomommmatsu@wd5.so-net.ne.jp

**Keywords:** hepatocellular carcinoma, lenvatinib, AFP, ALBI grade, bone metastasis

## Abstract

**Simple Summary:**

The first-line systemic therapy for advanced hepatocellular carcinoma (HCC) is atezolizumab plus bevacizumab therapy in many guidelines for HCC, however, there are no established therapeutic strategies in patients with intermediate stage and beyond the combination therapy. The aim of our retrospective study was to assess the real-world efficacy and safety of lenvatinib in Japanese patients with unresectable hepatocellular carcinoma. The results including median OS and PFS according to Barcelona Clinic Liver Cancer (BCLC) stage, liver function, and treatment history would be useful for making treatment strategies for patients with intermediate and beyond the combination therapy.

**Abstract:**

Background: Lenvatinib (LEN) has been approved for patients with unresectable hepatocellular carcinoma (u-HCC) since March 2018 in Japan. We performed a retrospective nationwide multicenter study to clarify the clinical characteristics of LEN in real-world practice. Methods: A total of 343 u-HCC patients who received LEN from March 2018 to May 2020 at 23 sites in Japan were registered. Results: During the median observation period of 10.5 months, 143 patients died. In Child-Pugh A (*n* = 276) and Child-Pugh B (*n* = 67) patients, the median overall survival (OS) was 21.0 and 9.0 months. The median progression-free survival (PFS) was 8.8 months in Child-Pugh A patients. The objective response rate (ORR) and disease control rate (DCR) according to modified response evaluation criteria in solid tumors (RECIST criteria) were 42.1% and 82.1%. The independent pretreatment factors associated with mortality in all patients were AFP ≥ 400 ng/mL (hazard ratio (HR) 2.00, 95% confidential interval (95% CI) 1.08–2.09, *p* < 0.0001), modified albumin-bilirubin (ALBI) grade 2b or 3 (HR 1.56, 95% CI 1.09–2.17, *p* = 0.012), major vascular invasion (HR 1.91, 95% CI 1.26–2.89, *p* = 0.0022), PS > 0 (HR 1.50, 95% CI 1.09–2.08, *p* = 0.014), and MTT (molecular targeted therapy) experience (HR 2.22, 95% CI 1.56–3.13, *p* = 0.00038). In the MTT naïve patients with ALBI grade 1 or modified ALBI 2a and BCLC stage B (*n* = 68), median OS and PFS were 25.3 and 12.3 months. Liver-related adverse events during LEN were the only significant adverse event associated with OS (HR 2.74, 95% CI 1.93–3.88, *p* < 0.0001). Among the Child-Pugh A patients with extrahepatic metastasis and no major vascular invasion, median PFS in the patients with bone metastasis was significantly shorter than those with lung or adrenal grand metastasis (6.3 vs. 12.5 months, *p* = 0.0025). Conclusion: LEN showed a high response rate in real-world practice. Pretreatment factors, including ALBI score, AFP, and major vascular invasion are important in making a treatment strategy for patients with u-HCC. The patients with bone metastasis would be candidates for new therapeutic approaches.

## 1. Introduction

Hepatocellular carcinoma (HCC) is the most common type of primary liver cancer and represents the third most common cause of cancer-related death [1]. For the treatment of unresectable hepatocellular carcinoma (u-HCC), tyrosine kinase inhibitors (TKIs), such as sorafenib [2,3] and regorafenib [4], have already been approved in many countries. Lenvatinib, an inhibitor of VEGFR1–3, fibroblast growth factor receptor (FGFR) 1–4, PDGFR-α, Ret, and kit, was reported non-inferior to sorafenib as first-line therapy for u-HCC in terms of overall survival (OS) [5]. In Japan, lenvatinib has been approved since Mar 2018 as a systemic agent for patients with u-HCC. Thereafter, lenvatinib has been widely used as a first-line treatment. As second-line agents for u-HCC, regorafenib, cabozantinib [6], nivolumab [7], and ramucirumab [8] have been approved in the USA. In Japan, regorafenib therapy has been performed as an established second-line therapy only for patients who tolerate sorafenib therapy, and ramucirumab was approved for patients with high AFP levels (≥400 ng/mL) in July 2019. Cabozantinib has been also used since November 2020. Nivolumab, and pembrolizumab [9] are not to be introduced for HCC treatment in Japan. In September 2020, atezolizumab plus bevacizumab [10] was approved for patients with u-HCC in Japan and would become first-line therapy. However, in the phase 3 trial of atezolizumab plus bevacizumab (IMbrave 150), only 15% of the patients were Barcelona Clinic Liver Cancer (BCLC) stage B, and there are no established biomarkers to make a therapeutic strategy for u-HCC except ramucirumab (AFP ≥ 400 ng/mL). Therefore, we conducted a retrospective nationwide study of lenvatinib to investigate the real-world data, including adverse events, and explore the clinical findings that would be useful for making a therapeutic strategy for u-HCC.

## 2. Methods

### 2.1. Study Design and Patients

#### 2.1.1. Study Design

This study was undertaken by the Japanese Red Cross Liver Study Group. It was a nationwide multicenter study including 23 centers of Japanese Red Cross Hospitals. All the patients treated with LEN between Mar 2018 and May 2020 in each institution were registered, and their outcomes were regularly followed up. There were no exclusion criteria, and all patients who provided informed consent were included. This study was conducted as per the ethical principles of the Declaration of Helsinki and the STROBE guidelines. The study was approved by the ethics committees of the Musashino Red Cross Hospital and each of the participating hospitals.

#### 2.1.2. Patients

The decision to start lenvatinib treatment was made by each local investigator based on the clinical guidelines in Japan. Every clinical decision was independently made by the local investigator at each center based on the official prescribing information accepted by the Japanese Ministry of Health, Labor and Welfare. After anonymization at the registry, data were collected at the central center (Musashino Red Cross Hospital). The information of each patient was collected, including sex, age, height, body weight, performance status accessed using the Eastern Cooperative Oncology Group scale, etiology of background liver disease, past experiences of trans-arterial chemo-embolization (TACE) and TKI therapy, tumor status, the presence of ascites or encephalopathy, laboratory data, and pretreatment tumor markers including alpha-fetoprotein (AFP) and Protein Induced by Vitamin K Absence or Antagonist-II. Liver function was accessed with the Child-Pugh classification [11], albumin-bilirubin (ALBI) grade [12], and modified ALBI (mALBI) grade [13]. ALBI grade 2 was classified as mALBI 2a or 2b using an ALBI score of −2.27 as the cut-off value. Clinical data after administration of LEN, the initial dose, dose reduction, drug interruption, drug discontinuation, treatment duration, and adverse events (AEs) were also reported. The liver was examined with CT or MRI by use of a triphasic scanning technique, and local investigators independently evaluated the tumors as per the modified response evaluation criteria in solid tumors (mRECIST) criteria [14]. The initial tumor assessments were done within 8 weeks of administering LEN therapy. AEs were evaluated as per the Common Terminology Criteria for Adverse Events version 4.0.

### 2.2. Statistics

The OS was measured from the date of LEN administration to the date of death from any cause. Patients who were lost to follow-up were censored at the last date they were known to be alive, and patients who remained alive were censored at the time of data cutoff. Progression-free survival (PFS) was measured from the date of LEN administration to the date of radiological tumor progression or death from any cause. Data are expressed as the median and range. Statistical analyses were performed using Fischer’s exact test, Mann–Whitney’s U test, a paired t test, Kaplan–Meyer method, and a log-rank test. A *p* value < 0.05 was considered to indicate statistical significance. All statistical analyses were performed using Easy R (EZR) version 1.29 (Saitama Medical Center, Jichi Medical University, Saitama, Japan), a graphical user interface for R (The R Foundation for Statistical Computing, Vienna, Austria) [15].

## 3. Results

### 3.1. Overall Efficacy and Safety Data

Between March 2018 and May 2020, 343 patients were registered. The baseline characteristics of the patients are shown in Table 1. In 109 patients who showed no hepatitis viral infection and without alcohol habit, the median body mass index was 24.6 (15.6–33.7). The percentage of obesity defined BMI ≥ 25 in Japan was 38.5% (*n* = 42). At the end of the data cut-off (30 September 2020), the median duration of follow-up was 10.5 (1.0–28.1) months. During the observation period, 143 patients died, and the median overall survival (OS) and progression-free survival (PFS) were 21.0 and 8.8 months in Child-Pugh A (*n* = 276) and 9.0 and 5.1 months in Child-Pugh B (*n* = 67) patients. According to modified RECIST, a radiological evaluation was performed in 280 patients, and the objective response rate (ORR) and disease control rate in patients who received radiological evaluation was 42.1% and 82.1%, respectively. According to modified RECIST criteria, complete response was noted in 16, partial response (PR) in 102, stable disease (SD) in 112, and PD in 50 patients. The median treatment duration was 4.97 (0.1–27.6) months. In Child-Pugh A patients, drug discontinuation was observed in 191 patients because of disease progression (*n* = 73), treatment-related AEs (TRAEs) (*n* = 102), worsening of other comorbidities (*n* = 15), and complete response (*n* = 1). In Child-Pugh B patients, drug discontinuation was observed in 47 patients because of disease progression (*n* = 9), TRAEs (*n* = 36), and worsening of other comorbidities (*n* = 2).

### 3.2. Overall Survival and Progression-Free Survival According to BCLC Stage, MTT Experience, and Modified ALBI Grade in Child-Pugh A Patients Treated with Lenvatinib

**Figure 1:**
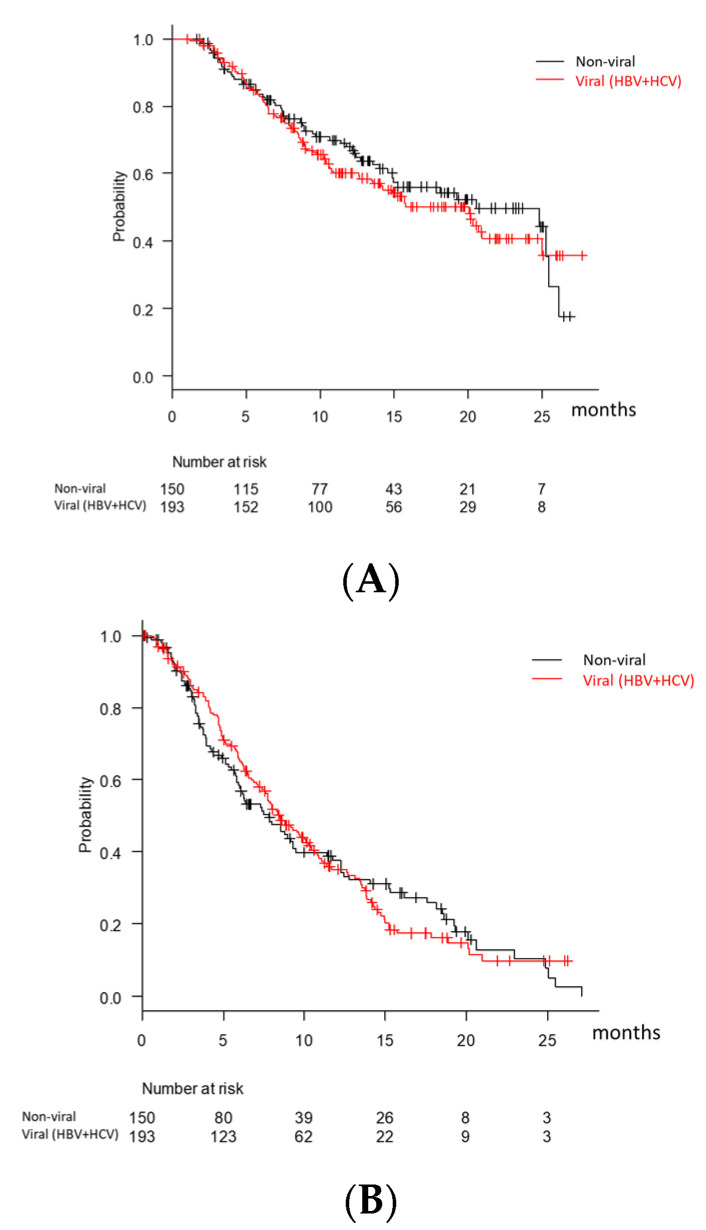
Overall survival (**A**) and progression-free survival (**B**) according to the etiology.

In this cohort, 97 of 343 (28.3%) patients had an experience of MTT. As the second-line therapy (after sorafenib), 51 patients received LEN, and one patient was treated with checkpoint inhibitors before LEN. Furthermore, 44 patients received LEN as the third-line (after sorafenib-regorafenib [*n* = 41], brivanib-sorafenib (*n* = 1), sorafenib-cabozantinib [*n* = 2]). One patient received LEN as the fourth-line after sorafenib, regorafenib, and cabozantinib. The comparison of the baseline characteristics of the MTT-naïve and experienced patients is shown in Table 2. The MTT-experienced patients showed younger age, lower albumin levels, higher ALBI scores, a higher rate of extrahepatic metastasis, and a lower incidence of MVI than the MTT-naïve patients. However, there were no differences in sex, total bilirubin, pretreatment AFP level between the two groups. The treatment duration of LEN was not significantly different between MTT naïve and experienced patients. The OS and PFS, according to BCLC stage, MTT experience, and ALBI grade in Child-Pugh A patients was shown in Table 3. The intrahepatic tumor status was beyond the up to seven criteria in 102 (77.3%) of 132 patients with BCLC stage B and Child-Pugh A. Additional TACE during LEN was performed in 27 patients to achieve CR or maintain DCR. Among them, median OS and PFS were 25.4 and 15.0 months. The statistical comparisons according to BCLC stage, MTT experience, and ALBI grade were shown in Table 4. There were no significant differences in OS and PFS, according to the etiology (viral vs. non-viral) (*p* = 0.48 and *p* = 0.88) (Figure 1A,B). We performed analyses about OS and PFS according to the inclusion criteria of the REFLECT study. In the REFLECT study [5], patients with 50% or higher liver occupation, obvious invasion of the bile duct, invasion at the main portal vein, or previous systemic therapy for HCC were excluded. The median OS and PFS in the patients (*n* = 174) who completely met the inclusion criteria of the REFLECT study [5] were 25.3 and 10.0 months, while the median OS and PFS in the patients (*n* = 71) who met the inclusion criteria except for the experience of previous systemic therapy for HCC were 15.2 and 7.1 months.

### 3.3. Overall Survival in Child-Pugh A Patients with Extrahepatic Metastasis and No Major Vascular Invasion

**Figure 2:**
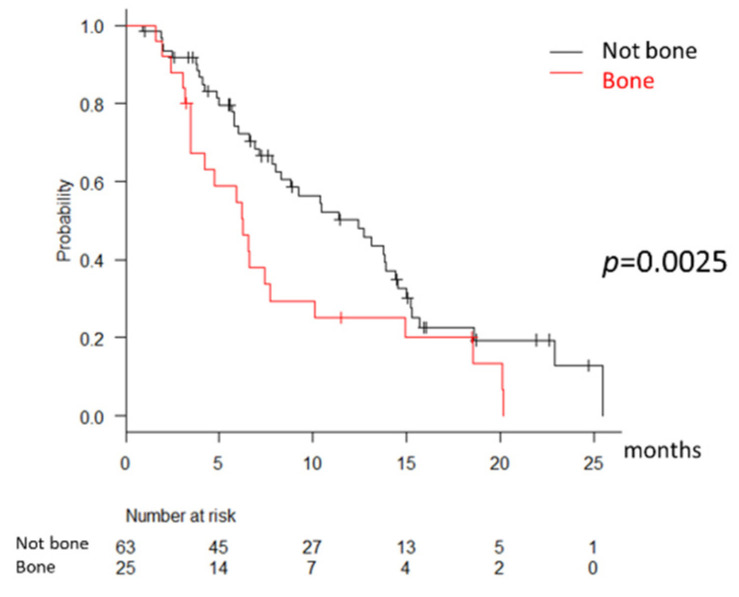
Progression-free survival according to the metastatic sites.

As extrahepatic metastatic sites, lung (*n* = 65), bone (*n* = 34), and adrenal gland (*n* = 19) were reported. In Child-Pugh A and MTT naïve patients (*n* = 88), the median PFS in the patients with bone metastasis and no MVI was significantly shorter than the patients with lung or adrenal gland metastasis and no MVI (6.3 vs. 12.5 months, *p* = 0.0025, Figure 2). The median OS between the two groups was not significantly different (20.2 vs. 25.5 months, *p* = 0.27). There were no significant differences in the pretreatment characteristics, including age, body weight, ALBI score, AST, and AFP level between the two groups.

### 3.4. Adverse Events (AEs) Associated with Lenvatinib Therapy

Major adverse events (>20%) during lenvatinib therapy were hypertension (*n* = 171, 49.9% (Grade ≥ 3 *n* = 14, 4.1%)), decreased appetite (*n* = 182, *n* = 53.1% (Grade ≥ 3 *n* = 9, 2.6%)), fatigue (*n* = 154, 44.9% (Grade ≥ 3 *n* = 14, 4.1%)), hand-foot skin reaction (*n* = 74, 21.6% (Grade ≥ 3 *n* = 3, 0.9%)), diarrhea (*n*-91, 26.5% (Grade ≥ 3 *n* = 3, 0.9%)), and liver-related AEs including increase AST, ALT, or T-Bil (*n* = 82, 23.9% (Grade ≥ 3 *n* = 14, 4.1%)). As the AEs associated with OS, decreased appetite and liver-related AEs were extracted by univariate analyses and liver-related AEs were the only significant AE associated with OS (HR 2.74, 95% CI 1.93–3.88, *p* < 0.0001) by a multivariate analysis. Pretreatment AST, ALBI score, and AFP level were significantly associated with liver-related AEs by univariate analyses and pretreatment AST level was the only significant factor associated with liver-related AEs (odds ratio 1.01, 95% CI 1.00–1.01, *p* = 0.043). By using a ROC analysis, we calculated the cut-off level of AST level and the patients with pretreatment AST ≥ 47 IU/mL had significantly higher risk for liver-related AEs (HR 3.5, 95% CI 2.08–5.92, *p* < 0.0001).

### 3.5. Factors Associated with Mortality in Lenvatinib Therapy

We analyzed the factors that were associated with mortality in all patients (Table 5) and separately between MTT naïve (Table 6) and experienced patients (Table 7) because the initial status at the first-line agent of the MTT experienced patients was different from that at the beginning of lenvatinib therapy. In the multivariate analysis, the independent pretreatment factors associated with mortality were AFP ≥ 400 ng/mL (hazard ratio (HR) 2.00, 95% confidential interval (95%CI) 1.08–2.09, *p* < 0.0001), ALBI grade 1 or modified ALBI grade 2a (HR 0.64, 95% CI 0.46–0.91, *p* = 0.012), major vascular invasion (HR 1.91, 95% CI 1.26–2.89, *p* = 0.0022), PS > 0 (HR 1.50, 95% CI 1.09–2.08, *p* = 0.014), and no MTT experience (HR 0.53, 95% CI 0.38–0.75, *p* = 0.00038) in all patients (Table 5).

### 3.6. Prognostic Model in Lenvatinib Therapy

**Figure 3:**
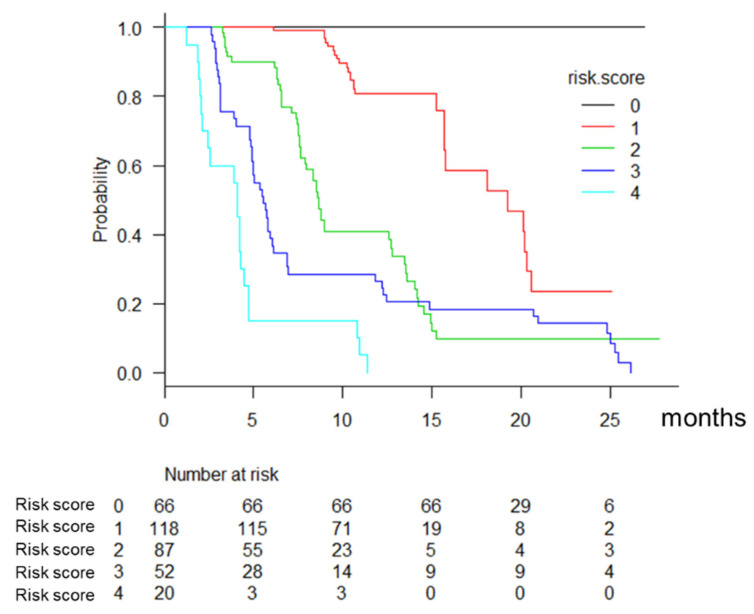
Overall survival according to the risk score.

We analyzed the survival probability using the scores calculated by the risk factors associated with mortality in lenvatinib therapy. Cox regression coefficients (β) were multiplied by a factor of 5 and round to the nearest unit to facilitate our prognostic point calculation. The final model was prognostic point = Performance Status (≤0 = 0, >0 = 1) + ALBI grade (ALBI grade 1 or modified ALBI 2a = 0, modified ALBI 2b or 3 = 1) + AFP (<400 ng/mL = 0, ≥400 ng/mL = 1) + Major vascular invasion (No = 0, Yes = 1) + MTT experience (No = 0, Yes = 1). Based on the Youden index, a point of 2.0 was calculated as the cut-off for categorizing patients as being at low-risk (≤2) or high-risk (>2) of poor overall survival (<12 months) after lenvatinib therapy, and the cut-off revealed a sensitivity of 71.9% and specificity of 86.1%. The area under the receiver operating characteristic curve (AUC) of the model was 0.859 (95% CI: 0.821–0.897). No patient with a risk score of 0 (*n* = 66) died during the observation period, and the median OS in patients with risk score of 1 (*n* = 118) was 19.2 months (Figure 3).

## 4. Discussion

To our knowledge, this is the first study to show the median OS and PFS clearly according to BCLC stage, ALBI grade, and MTT experience, and the difference in PFS according to the metastatic sites. This cohort included patients who were not eligible for the phase 3 clinical trial [5], and the results reflected the real situation of LEN therapy in clinical practice. The present results showed that the efficacy or safety was similar to that of the phase 3 trial [5], and there were no unknown AEs in actual clinical practice. In this study, we identified the factors that had a significant association with mortality in patients who were given LEN therapy; this finding would contribute to the decision-making in patients with unresectable HCC.

In the phase 3 trial of LEN [5], LEN showed significantly better PFS, time to progression, and objective response than sorafenib, with a comparable OS. In our study, 201 patients treated with LEN were MTT naïve and Child-Pugh A. The median OS and PFS in these naïve patients were 47.3% and 86.3%, respectively. In the REFLECT study [5], the ORR and DCR were 24.1% and 75.5%, respectively, as per the investigator review as per the mRECIST criteria. Although the median age of our patients was almost 10 years more than that of the patients in the phase 3 study [5], the high response rates to first-line therapy were confirmed in actual clinical practice. The percentage of BCLC stage B HCC in our cohort was 47% in MTT naïve patients, while the percentage in the phase 3 study (lenvatinib group) [5] was 22%. A major reason for prolonged survival in our cohort was associated with the difference in the initial tumor status. The standard therapy for BCLC stage B HCC is TACE in Japan, as well as most countries, and 87.0% of the BCLC stage B patients in our study received TACE before lenvatinib therapy. The median TACE cycles before lenvatinb therapy were 2.5 (0–12). Most patients were diagnosed with TACE failure or refractory. The median PFS and time of tumor progression (TTP) in Child-Pugh B patients were 5.1 and 5.8 months.

Sorafenib has been used since 2009 in Japan, and regorafenib (2017) and ramucirumab (2019) have been approved as second-line therapy for u-HCC. Ramucirumab can be administered only in patients with high AFP levels (≥400 ng/mL). Pembrolizumab has been approved only for patients with high microsatellite instability (MSI) since Dec 2018. However, some previous reports [16,17,18] have revealed that the proportion of patients with MSI-high HCC was deficient (0–8%). Nivolumab for HCC was not introduced in Japan. Cabozantinib was approved in November 2020 and there is a small number of patients treated with cabozantinib in Japan. Atezolizumab plus bevacizumab has been approved since September 2020 in Japan. It would become the most recommended first-line therapy for u-HCC because the combination therapy, including a checkpoint inhibitor, revealed superior OS and PFS compared to sorafenib in the phase 3 trial [10]. However, in the phase 3 trial, almost 85% of the patients were BCLC stage C, and the patients with intermediate-stage HCC were only 15%. According to our results, the median OS and PFS in patients with MTT naïve, Child-Pugh A, and BCLC stage B (*n* = 97) were 25.2 and 9.7 months. Furthermore, the MTT naïve patients with BCLC stage B and good liver function (ALBI grade 1 or modified ALBI 2a) showed excellent median OS and PFS (25.3 and 12.3 months). Prospective studies including many patients at multi centers, are necessary to make a therapeutic strategy for intermediate-stage HCC.

One of the most important findings in our study was that patients with bone metastasis had significantly shorter PFS than patients with lung or adrenal grand metastasis (6.3 vs. 12.5 months, *p* = 0.0025). Jiao, et al. [19] recently reported the differences in the tumor microenvironment in patients with metastatic castration-resistant prostate cancer and bone metastasis. Bollig CA, et al. [20] reported that polymetastatic disease and bone metastasis were associated with worse prognosis, independent of treatment received in patients with head and neck cancer. Moreover, Haaker L, et al. [21] reported that the presence of bone metastasis was an unfavorable prognostic factor associated with shorter PFS and OS in metastatic papillary renal cell carcinoma patients treated with vascular endothelial growth factor receptor tyrosine kinase inhibitors. Further studies both in vitro and vivo should be performed to explain the mechanism and improve clinical outcomes in patients with bone metastasis. Harding JJ, et al. [22] reported the presence or absence of bone metastasis at the presentation of extrahepatic disease in HCC patients had no impact on OS; however, skeletal-related events (SREs) were associated with worse OS. In their study, the unadjusted HRs for bisphosphonates and sorafenib therapy were 0.3 (0.13–0.64; *p* < 0.01) and 0.4 (0.19–0.77; *p* < 0.02), and no other disease-specific factors modulated the chance of developing SREs. In our study, there were no data about SREs and further information is necessary to evaluate our results.

As significant factors associated with OS, pretreatment performance status, liver function, AFP level, MVI, and no MTT experience were extracted in a multivariate analysis. Previous reports [23,24,25] revealed that pretreatment liver function based on Child-Pugh score and ALBI score was the most important factor associated with OS, PFS, and treatment discontinuation due to AEs in LEN therapy. We reported that relative dose intensity (RDI) of LEN was associated with clinical outcome in lenvatinib therapy [26], and the recent study [27] showed the usefulness of weekend-off lenvatinib to maintain RDI and avoid treatment discontinuation due to AEs. We also made a prognostic model in lenvatinib therapy, which consisted of five factors, including pretreatment PS, modified ALBI grade, AFP, major vascular invasion, and MTT experience. In the high-risk group (risk score > 2, *n* = 72), median OS was 4.9 months, while no patient with a risk score of 0 died during the observation period. By using this model clinicians would be able to make a therapeutic strategy confidently.

There are certain limitations to our study. The study was retrospective, and tumor response was not evaluated in all patients. In our study, 63 (18%) patients could not be evaluated with dynamic CT or MRI because of renal dysfunction or worsening general condition. All therapeutic decisions, including the initial dose, dose modification, interruption duration, and post-LEN therapies were taken individually by each investigator.

To our knowledge, our report is the first study to reveal the median OS and PFS clearly according to pretreatment factors, and the difference in clinical outcome according to the existence of bone metastasis. Recently, many molecular targeted agents have been approved for u-HCC, and by considering pretreatment factors including liver function (ALBI score), AFP level, and MVI we can construct useful therapeutic strategies for patients with unresectable HCC. Performing effective sequential therapies provides better survival in patients with u-HCC.

## 5. Conclusions

Lenvatinib therapy in patients with unresectable HCC showed a high response rate in real-world practice similar to the phase 3 trial. Pretreatment factors, including ALBI score, AFP and major vascular invasion, and MTT experience, were associated with OS after the administration of LEN. The patients with bone metastasis would be candidates for new therapeutic approaches.

## Figures and Tables

**Table 1:** Baseline characteristics of the patients.

	*n* = 343
Age (years), median (range)	73 (44–93)
Sex: Male/Female (%)	277 (81)/66 (19)
MTT naïve/experienced	246 (72)/97(28)
Body weight (kg): median (range)	60.0 (30.3–101)
Etiology HBV/HCV/Alcohol/Others (%)	49 (14)/144 (42)/41 (12)/109 (32)
Child–Pugh A/B (%)	276 (80)/67 (20)
Pretreatment ALBI score: median (range)	−2.34 (−0.60 to −3.52)
Modified ALBI grade 1/2a/2b/3 (%)	95 (28)/98 (29)/136 (39)/14 (4)
ECOG PS 0/1/2 (%)	226 (66)/110 (32)/7 (2)
BCLC stage A/B/C (%)	9 (3)/152 (44)/182 (53)
Major vascular invasion Yes/No (%)	60 (17)/283 (83)
Extrahepatic metastasis Yes/No (%)	118 (34)/225 (66)
Baseline AFP concentration (ng/mL), median (range)	75.1 (1.2–458151)
Baseline AFP < 400 ng/mL Yes/No/unknown (%)	224 (65)/104 (30)/15 (5)

Abbreviations: MTT, molecular targeted therapy; HBV, hepatitis B virus; HCV, hepatitis C virus; ALBI sore, albumin-bilirubin score; ECOG, Eastern Cooperative Oncology Group; BCLC stage, Barcelona Clinic Liver Cancer stage; AFP, alpha-fetoprotein.

**Table 2:** Comparison of the baseline characteristics between MTT naïve and experienced patients.

	MTT Naïve (*n* = 246)	MTT Experienced (*n* = 97)	*p*-Value
Age (median)	74	70	0.0016
Gender (male, %)	194 (79)	83 (86)	0.17
BW (median, kg)	60.5	60.0	0.17
Alb (median, g/dL)	3.7	3.4	0.0028
T-Bil (median, mg/dL)	0.8	0.8	0.37
ALBI score (median)	−2.36	−2.22	0.017
pretreatment AFP (median, ng/mL)	56	170	0.13
Extrahepatic metastasis (yes, %)	75 (30)	43 (44)	0.023
Major vascular invasion (yes, %)	50 (20)	10 (10)	0.028
Treatment duration of lenvatinib (median, day)	125	172	0.6

Abbreviations: MTT, molecular targeted therapy; BW, body weight; Alb, albumin; T-Bil, total bilirubin; ALBI sore, albumin-bilirubin score; AFP, alpha-fetoprotein.

**Table 3:** The median OS and PFS according to BCLC stage, MTT experience, and ALBI or modified ALBI grade in Child-Pugh A patients.

	Median OS (Months)	Median PFS (Months)
BCLC stage B, MTT naïve, ALBI 1 or mALBI 2a (*n =* 68)	25.3	12.3
BCLC stage B, MTT naïve, mALBI 2b (*n =* 29)	13.5	7.7
BCLC stage B, MTT experienced, ALBI 1 or mALBI 2a (*n =* 14)	15.2	11.8
BCLC stage B, MTT experienced, mALBI 2b (*n =* 21)	14.0	5.5
BCLC stage C, MTT naïve, ALBI 1 or mALBI 2a (*n =* 64)	20.9	12.7
BCLC stage C, MTT naïve, mALBI 2b (*n =* 32)	15.2	6.6
BCLC stage C, MTT experienced, ALBI 1 or mALBI 2a (*n =* 30)	9.7	7.1
BCLC stage C, MTT experienced, mALBI 2b (*n =* 9)	*	6.8

* not reached. Abbreviations: MTT, molecular targeted therapy; ALBI, albumin-bilirubin score; BCLC stage, Barcelona Clinic Liver Cancer stage.

**Table 4:** The statistical comparisons according to BCLC stage, MTT experience, and ALBI grade.

			*p*-Value	
			OS	PFS
MTT naïve	BCLC stage B	ALBI 1 or mALBI 2a vs. mALBI 2b	0.009	0.002
MTT naïve	BCLC stage C	ALBI 1 or mALBI 2a vs. mALBI 2b	0.136	0.036
MTT experienced	BCLC stage B	ALBI 1 or mALBI 2a vs. mALBI 2b	0.31	0.034
MTT experienced	BCLC stage C	ALBI 1 or mALBI 2a vs. mALBI 2b	0.13	0.96
ALBI 1 or mALBI 2a	MTT naïve	BCLC stage B vs. BCLC stage C	0.014	0.1
ALBI 1 or mALBI 2a	MTT experienced	BCLC stage B vs. BCLC stage C	0.22	0.12
mALBI 2b	MTT naïve	BCLC stage B vs. BCLC stage C	0.76	0.91
mALBI 2b	MTT experienced	BCLC stage B vs. BCLC stage C	0.14	0.4

Abbreviations: MTT, molecular targeted therapy; ALBI, albumin-bilirubin score; BCLC stage, Barcelona Clinic Liver Cancer stage.

**Table 5:** Factors associated with mortality in lenvatinib therapy (All patients).

	Univariate	Multivariate		
Factor	*p* Value	HR	95% CI	*p* Value
Age (years)	0.16			
Body weight (kg)	0.62			
Performance Status > 0	0.015	1.50	1.09–2.08	0.014
Pretreatment modified ALBI grade 2b or grade 3	0.00027	1.56	1.09–2.17	0.012
Pretreatment AFP ≥ 400 ng/mL	<0.0001	2.00	1.42–2.80	<0.0001
Extrahepatic metastasis	0.52			
Major vascular invasion	0.00089	1.91	1.26–2.89	0.0022
MTT experience	0.00043	2.22	1.56–3.13	0.00038

**Table 6:** Factors associated with mortality in lenvatinib therapy (MTT naïve patients).

	Univariate	Multivariate		
Factor	*p* Value	HR	95% CI	*p* Value
Age (years)	0.044			0.27
Body weight (kg)	0.41			
Performance Status > 0	0.45			
Pretreatment modified ALBI grade 2b or grade 3	0.0005	1.92	1.25–2.96	0.0032
Pretreatment AFP ≥ 400 ng/mL	0.0001	2.05	1.32–3.18	0.0014
Extrahepatic metastasis	0.80			
Major vascular invasion	0.0001	1.89	1.15–3.08	0.012

**Table 7:** Factors associated with mortality in lenvatinib therapy (MTT experienced patients).

	Univariate	Multivariate		
Factor	*p* Value	HR	95% CI	*p* Value
Age (years)	0.12			0.056
Body weight (kg)	0.042			
Performance Status > 0	0.00078	2.36	1.41–3.92	0.0009
Pretreatment modified ALBI grade 2b or grade 3	0.29			
Pretreatment AFP ≥ 400 ng/mL	0.0056	2.39	1.37–4.16	0.0020
Extrahepatic metastasis	0.99			
Major vascular invasion	0.42			

Abbreviations: MTT, molecular targeted therapy; ALBI grade, albumin-bilirubin grade; AFP, alpha-fetoprotein.

## Data Availability

The data presented in this study are available on request from the corresponding author.
